# Coupling of Cell Surface Biotinylation and SILAC-Based Quantitative Proteomics Identified Myoferlin as a Potential Therapeutic Target for Nasopharyngeal Carcinoma Metastasis

**DOI:** 10.3389/fcell.2021.621810

**Published:** 2021-06-09

**Authors:** Maoyu Li, Fang Peng, Guoqiang Wang, Xujun Liang, Meiying Shao, Zhuchu Chen, Yongheng Chen

**Affiliations:** ^1^Department of Gastroenterology, Xiangya Hospital, Central South University, Changsha, China; ^2^NHC Key Laboratory of Cancer Proteomics, Xiangya Hospital, Central South University, Changsha, China; ^3^Key Laboratory of Medical Genetics and College of Life Sciences, Central South University, Changsha, China

**Keywords:** nasopharyngeal carcinoma, cell surface proteins, metastasis, biotinylation, SILAC

## Abstract

Distant metastasis is a major cause of treatment failure in nasopharyngeal carcinoma (NPC) patients. Cell surface proteins represent attractive targets for cancer diagnosis or therapy. However, the cell surface proteins associated with NPC metastasis are poorly understood. To identify potential therapeutic targets for NPC metastasis, we isolated cell surface proteins from two isogenic NPC cell lines, 6-10B (low metastatic) and 5-8F (highly metastatic), through cell surface biotinylation. Stable isotope labeling by amino acids in cell culture (SILAC) based proteomics was applied to comprehensively characterize the cell surface proteins related with the metastatic phenotype. We identified 294 differentially expressed cell surface proteins, including the most upregulated protein myoferlin (MYOF), two receptor tyrosine kinases(RTKs) epidermal growth factor receptor (EGFR) and ephrin type-A receptor 2 (EPHA2) and several integrin family molecules. These differentially expressed proteins are enriched in multiple biological pathways such as the FAK-PI3K-mTOR pathway, focal adhesions, and integrin-mediated cell adhesion. The knockdown of MYOF effectively suppresses the proliferation, migration and invasion of NPC cells. Immunohistochemistry analysis also showed that MYOF is associated with NPC metastasis. We experimentally confirmed, for the first time, that MYOF can interact with EGFR and EPHA2. Moreover, MYOF knockdown could influence not only EGFR activity and its downstream epithelial–mesenchymal transition (EMT), but also EPHA2 ligand-independent activity. These findings suggest that MYOF might be an attractive potential therapeutic target that has double effects of simultaneously influencing EGFR and EPHA2 signaling pathway. In conclusion, this is the first study to profile the cell surface proteins associated with NPC metastasis and provide valuable resource for future researches.

## Introduction

Nasopharyngeal carcinoma (NPC) is an aggressive malignancy common in Southern China and Southeast Asia. NPC is prone to early metastasis, but patients are asymptomatic at the early stage (Chua et al., 2016). Therefore, most NPC patients (60–70% cases) are diagnosed at an advanced stage (Mao et al., 2009). At present, radiotherapy and chemo-radiotherapy are the routine treatment strategies for patients with non-metastatic or locally advanced NPC. It has been reported that for approximately 25-30% of patients therapy still fails, with local recurrence and/or distant metastases, and the prognosis for patients with metastatic disease is poor (Lai et al., 2011). NPC metastasis and recurrence are the main bottleneck for treatment. Therefore, the demand for new therapeutic strategies for advanced NPC, to further improve treatment outcome is urgent.

The interaction between cancer cells and surrounding stromal cells in the tumor microenvironment plays a key role in cancer cell migration, invasion and metastasis. Cell surface proteins are important player in these processes. Cell surface proteins, primarily represented by plasma membrane proteins, account for approximately one-third of the proteins encoded by the human genome (Vuckovic et al., 2013). These proteins have a variety of important biological functions, such as signal transduction, cell-to-cell recognition, and material transport. Given their accessibility and significant biological function, they have become ideal targets for novel therapies. At present, more than two-thirds of the current protein-based drug targets are plasma membrane proteins (Overington et al., 2006).

Due to their low abundance and poor solubility, routine proteomics analysis strategies are insufficient for identification and characterization of these proteins. Therefore, strategies combining cell surface enrichment and quantitative proteomics, which can reduce sample complexity, resulting in identification of more intermediate to low abundant proteins, have been widely exploited. Several methods, such as ultracentrifugation, cell-surface biotinylation and cell surface capture, have been used to extract or enrich the cell surface and/or plasma membrane proteins. Cell-surface biotinylation is one of the most commonly used methods for isolation of plasma membrane proteins, in which the extracellular domains of integral and plasma membrane-associated proteins are covalently labeled with a reactive biotin ester. Through tagging with a membrane-impermeable biotin, cell surface proteins can be separated from cell extracts with streptavidin-linked beads. This approach has been widely used to separate plasma membrane proteins associated with tumor metastasis (Lund et al., 2009), tumor cell cycle (Özlü et al., 2015), and T cell-mediated autoimmunity (Buehler et al., 2018), which demonstrates that it can improve throughput and selectivity for detection of membrane proteins in low abundance.

However, the cell surface proteins involved in NPC metastasis have not been well investigated. Here, Stable isotope labeling by amino acids in cell culture (SILAC) was employed to compare cell surface proteins enriched via surface biotinylation from a pair of isogenic NPC cells with low and high metastatic potential. A total of 1029 proteins localized at the cell surface were identified. Of the quantified cell surface proteins, 294 differentially expressed proteins were enriched in multiple pathways such as the phosphatidylinositol 3-kinase - protein kinase B (PI3K-AKT) signaling pathway, integrin pathway. We verified that the most upregulated membrane protein myoferlin (MYOF) was metastasis-related protein whose knockdown suppressed the malignant phenotype of NPC tumor cells. We further, for the first time experimentally confirmed that the interactions of MYOF with epidermal growth factor receptor (EGFR) and ephrin type-A receptor 2 (EPHA2), both of which are metastasis-related receptor tyrosine kinases (RTK). We found that MYOF impairs not only EGFR activation and its downstream epithelial–mesenchymal transition (EMT) process, but also EPHA2 ligand-independent activation. Therefore, MYOF targeted therapy might have double effect that simultaneously influence EGFR and EPHA2 pathway in NPC. Collectively, this is the first large-scale characterization of NPC metastasis-related cell surface membrane proteins, which provide valuable resource for future researches.

## Materials and Methods

### Cell Culture and Small Hairpin RNA (shRNA) Lentiviral Transduction

A isogenic pair of human malignant NPC cell lines, 5-8F (high metastatic potential) and 6-10B (low metastatic potential), were cultured in Roswell Park Memorial Institute 1640 (RPMI-1640) medium (Gibco BRL, Grand Island, NY, United States) supplemented with 10% fetal bovine serum (FBS) (Gibco) at 37°C in 5% CO_2_.

For lentiviral transduction, cells were trypsinized and resuspended in RPMI-1640 containing 10% FBS at a density of 1 × 10^6^ cells/ml. pLV-EGFP-T2A-Neo-U6-MYOF-shRNA or pLV-EGFP-T2A-Neo-U6-scramble-shRNA (Cyagen) was added to the trypsinized cell suspension at a multiplicity of infection (MOI) of 30. Cells were subjected to puromycin selection to generate stable MYOF-knockdown cell lines.

### SILAC Labeling

For SILAC experiments, RPMI-1640 medium lacking L-lysine, [^13^C_6_,^15^N_2_]-L lysine/[^13^C_6_,^15^N_4_]-L Arginine (heavy label) and unlabeled L-lysine/L-Arginine (light label) were purchased form Thermo Scientific (Baltimore, MD, United States). Light and heavy RPMI-1640 media were prepared according to the standard RPMI-1640 formulation except with the supplement of dialyzed FBS (Invitrogen). The low metastatic cell line were grown in light medium and the high metastatic 5-8F cells were cultured in heavy medium for at least 6 divisions to allow complete incorporation of the isotope-labeled amino acids. These experiments were performed in triplicate.

### Affinity Purification of Cell-Surface Membrane Proteins

Cell surface protein isolation was performed according to the manufacturer’s instructions. Briefly, after cells attached to the flasks, they were washed three times with PBS (pH 7.4) at 37°C to remove culture medium and FBS. A 1-mL solution of 0.5 mg/mL Sulfo-NHS-SS-biotin (Pierce, Rockford, IL, United States) in PBS was added to each flask, and the cells were incubated at 37°C for 10 min. Then, 1 mL of quenching solution was added to each flask and incubated with cells at room temperature for 5 min to quench the reaction, and then, the cells were washed with ice-cold PBS three times to remove excess reagent and byproducts. Biotinylated 6-10B and 5-8F cells were harvested into ice-cold PBS and then pooled in a 1:1 ratio. The mixed cells were lysed on ice for 30 min with gentle shaking. Cell lysates were centrifuged at 15000 × *g* for 30 min at 4°C, and the supernatants were collected. Avidin-agarose resin was added into spin columns and washed with washing buffer three times. The clarified cell lysates were placed into the spin columns. After a 60-min incubation at room temperature with end-over-end mixing, the resin was washed three times. The bound proteins were eluted with 50 mM DTT in SDS loading buffer. The elution process was repeated, and the eluates were combined and stored at −20°C for further analysis.

### Enzymatic Digestion of Proteins

The eluted proteins were reduced with dithiothreitol (DTT) at a final concentration of 10 mM for 30 min at room temperature and then alkylated with iodoacetamide (IAA) at a final concentration of 50 mM for 30 min in the dark. The resulting solutions were diluted 10 times with 100 mM NH_4_HCO_3_ (pH 8.5) and digested with mass spectrometry (MS)-grade trypsin (Promega, Madison, WI, United States) at an enzyme-to-substrate ratio of 1/75 (w/w) at 37°C overnight.

### MS and Data Acquisition

Peptide samples were analyzed in duplicate (two technical replicates). For each technical replicates, the peptide mixtures were fractionated into 5 or 10 fractions via strong anion exchange chromatography according to published procedures (Li et al., 2016). Each fraction was independently analyzed using an EASY-nano LC system (Proxeon Biosystems, Odense, Denmark) coupled online with an LTQ-Orbitrap Velos mass spectrometer (Thermo Fisher Scientific, Waltham, MA, United States). Briefly, peptides were loaded onto a PepMap C18 trap column (75 μm, 15 cm; Dionex Corp.) and eluted using a gradient from 100% solvent A (0.1% formic acid) to 35% solvent B (0.1% formic acid, 100% acetonitrile) for 38 min, 35 to 90% solvent B for 15 min, and 100% solvent B for 5 min (a total of 65 min at 200 nL/min). After each run, the column was washed with 90% solvent B and re-equilibrated with solvent A. Mass spectra were acquired in positive ion mode applying data-dependent automatic survey MS scan and tandem mass spectra (MS/MS) acquisition modes. Each MS scan in the Orbitrap analyzer (mass range = m/z 350–1800, resolution = 100,000) was followed by MS/MS of the seven most intense ions in the LTQ. Fragmentation in the LTQ was performed via high-energy, collision-activated dissociation, and selected sequenced ions were dynamically excluded for 30 s.

### MS Data Processing and Quantitative Analysis

Raw MS files were processed using MaxQuant (version 1.5.8.30) (Cox and Mann, 2008). Carbamidomethylation (C) was set as a fixed modification, and oxidation (M), deamidation (N), N-acetyl (N-term), and DSP (K, N-term) were used as variable modifications. Missed tryptic cleavage was set to 2, and the minimal length required for a peptide was 6 amino acids. The tolerance of the precursor mass was 10 ppm, and the fragment mass tolerance was set to 0.5 Da. The false discovery rates (FDRs) for peptide and protein identification were both set to 0.01. All other parameters were set to the default settings. The datasets were searched against the UniProt human database (version 2019.07.06). Labeling was set to doublets of K0R0 and K8R10. For protein quantification, razor and unique peptides were used with two or more ratio counts. The protein SILAC ratio was calculated as the median of all SILAC peptide ratios.

Further analyses were performed using Perseus software (Tyanova et al., 2016). At least one unique peptide in at least one of the two technical replicates for each biological replicate was required for successful protein identification. Proteins that were identified in at least two biological replicates were selected for quantification analysis. Protein quantification was calculated by averaging the SILAC ratios of all replicate experiments. One-sample *t*-test was performed to evaluate the significance of the ratio. For calculation of correlation coefficients, datasets were filtered for entries with valid quantifications in all experiments. Differentially expressed protein groups were defined as those that had expression ratios larger than twofold and a *p*-Value < 0.05. Plasma membrane, extracellular, and cell surface proteins were annotated based on the GO (Gene Ontology) category Cellular compartment (CC) annotation using Perseus software.

### Cell Proliferation Assay

The 5-8F cells were plated at 2 × 10^3^ cells per well in 96-well tissue culture plates and cultured in 10% FBS complete RPMI-1640 medium for 6 days. Every 24 h, 20 μl of CCK8 reagent (5 mg/ml; Beyotime) was added to the wells, and cells were further incubated for 2 h. The absorbance of each well was read with a Bio-Tek Instruments EL310 Microplate Autoreader at 450 nm. Experiments were performed in triplicate.

### Wound Healing Assay

Cell migration was determined with a scratch wound healing assay. Briefly, cells were grown to confluence in RPMI-1640 medium containing 10% FBS overnight in a 6-well plate. Cell monolayers were wounded by dragging a pipette tip through them. Cells were washed to remove cellular debris and allowed to migrate for 24-48 h. Images were taken at 0, 24, and 48 h after wounding under an inverted microscope.

### Cell Migration Assay

Migration activity was measured with a Transwell assay (Corning, 3422). Approximately 5 × 10^4^ cells were added to the upper chamber in 200 μl of 1% FBS RPMI-1640 medium. The lower chamber contained 500 μl of 10% FBS complete RPMI-1640 medium. The plates were incubated for 24 h at 37°C in 5% CO_2_. Cells were fixed in 3.7% formaldehyde solution for 15 min and stained with 0.05% crystal violet in PBS for 15 min. Cells on the upper side of the filters were removed with cotton-tipped swabs. Cells on the underside of the filters were examined and counted under a microscope. Each clone was plated in triplicate for each experiment, and each experiment was repeated three times.

### Cell Invasion Assay

Invasion assays were performed in 24-well, 8-mm pore size Transwell chambers precoated with Matrigel (Corning) according to the manufacturer’s instructions. The upper chamber was filled with 1 × 10^5^ cells in RPMI-1640 medium containing 0.5% FBS. The lower chamber was filled with RPMI-1640 medium containing 10% FBS as a chemoattractant. The plates were incubated for 48 h at 37°C in 5% CO_2_. Cells were fixed in 3.7% formaldehyde solution for 15 min and stained with 0.05% crystal violet in PBS for 15 min. Cells on the upper side of the filters were removed with cotton-tipped swabs. Cells on the underside of the filters were examined and counted under a microscope. Each clone was plated in triplicate for each experiment, and each experiment was repeated at least three times.

### EGF-Mediated EMT Assay

The EGF-inducible EMT assay was performed according to previously described methods with minor modifications (Yip and Seow, 2012). Briefly, shMYOF-transfected 5-8F cells were trypsinized and plated at a density of 200,000/well in a 6-well plate. The culture medium was supplemented with EGF at a final concentration of 20 ng/mL. Cells were cultured further for 24 to 48 h. The expression levels of EMT markers were detected via western blotting.

### Antibodies and Western Blotting

For immunostaining or western blotting, primary antibodies against the following proteins were used: MYOF (Santacruz); EGFR, pEGFR, EPHA2, and EPHA2-pS897 (Cell Signaling); VIM and E-cadherin (Abcam); and actin (Pierce). For western blot analysis, cells were washed once with ice-cold PBS and then lysed in cold RIPA buffer supplemented with protease inhibitor cocktail (Roche Applied Biosciences). Cell lysate was centrifuged at 12,000 × *g* for 30 min, boiled in 2 × loading sample buffer, separated on a 10% gradient SDS–PAGE gel and blotted onto a PVDF membrane. The membrane was blocked with 0.1% Tween-20 in TBS containing 5% non-fat milk and probed with the indicated primary antibody, followed by incubation with the appropriate secondary antibody conjugated to horseradish peroxidase (Sigma), and the signals were visualized via ECL (Millipore).

### Co-immunoprecipitation (Co-IP)

The Co-IP experiment was performed as previously described (Sun et al., 2007). Briefly, cells were lysed in lysis buffer (20 mM Tris, pH 7.5, 150 mM NaCl, 1 mM EDTA, 5 mM DTT, 1% NP-40, 1 mM Na_3_VO_4_, 1 mM PMSF) for 60 min on ice, followed by centrifuging at 12000 × *g* for 30 min at 4°C to remove cell debris. The whole cell lysates were precleared by incubation with Protein A/G Plus-Agarose (Santa Cruz Biotechnology) for 1 h at 4°C. The clarified supernatants were incubated with the indicated antibody or control (non-immune) serum overnight at 4°C, followed by incubation with Protein A/G Plus-Agarose (Santa Cruz Biotechnology) at 4°C for another 2 h. The agarose was washed 6 times and boiled in 2 × sodium dodecyl sulfate sample buffer for 5 min. The immunoprecipitated complexes were resolved using sodium dodecyl sulfate polyacrylamide gel electrophoresis and immunoblot with specific antibodies.

### Total Cellular Protein and Membrane Fractionation

Cells were harvested in homogenization buffer A [20 mM TBS with 1 mM PMSF and complete EDTA-free protease inhibitor mixture (Roche)]. The cell suspension was homogenized 10 times on ice and then briefly centrifuged at 2,000 × *g* for 5 min at 4°C. The clarified lysate was then centrifuged at 80,000 rpm using a Beckman Ultracentrifuge (Beckman Coulter) for 1 h at 4°C. The membrane pellet was washed twice with buffer A and then centrifuged at 80,000 × *g* for 30 min at 4°C. The membrane pellet was dissolved in RIPA and stored in a refrigerator for further analysis.

### Immunohistochemistry (IHC)

Fifty paraffin-embedded NPC tumor tissue specimens were collected from the archives in the Pathology Department (Xiangya Hospital, Central South University). IHC was performed according to the procedure described in our previous study (Xiao et al., 2010). Briefly, the sections were incubated with primary antibody overnight at 4°C and then with biotinylated secondary antibody, followed by addition of avidin-biotin peroxidase. Diaminobenzidine was used as the chromogen. The immunostaining intensity was evaluated as previously described with minor modification (Li et al., 2016). Briefly, each specimen was scored according to the intensity of staining and the area of staining. The intensity of staining was graded as follows: 0, no staining; 1 +, mild staining; 2 +, moderate staining; 3 +, intense staining. The area of staining was scored as follows: 0, no staining of cells in any microscopic field; 1 +, <30% of tumor cells stained positively; 2 +, between 30 and 60% stained positively; 3 +, >60% stained positively. A combined staining score (intensity + area, ranging from 0 to 6) was obtained for each case. A combined staining score of ≤ 4 was considered weak staining (low expression); a score > 4 was considered strong staining (high expression).

### Bioinformatic Analysis

Differentially expressed proteins (DEPs) were subjected to Gene set enrichment analysis (GSEA) using GSEAPreranked tools in GSEA software (version 3.0) to gain insight into over-represented Wikipathways^1^. The significance of enrichment was set to *p* < 0.05. Protein-protein interactions and their first neighbors for DEPs were retrieved using Harmonizome^2^ to query Pathway Commons Protein-Protein Interactions dataset and imported into Cytoscape for visualization or presentation (Rouillard et al., 2016). Pathvisio (version 3.3^3^) was used to visualize the ratio values onto biological pathways obtained from Wikipathway. Student’s *t*-test was used to analyze the significance of differences between groups. Correlations between MYOF expression and clinical characteristics associated with NPC tissues were analyzed with a Fisher’s exact test. A *p*-Value < 0.05 was considered statistically significant. The Cancer Cell Line Encyclopedia(CCLE) proteomic data download from the website^4^ (Nusinow et al., 2020).

## Results

### Proteomics Analysis of Low and High Metastatic NPC Cell Surface Proteins

**FIGURE 1:**
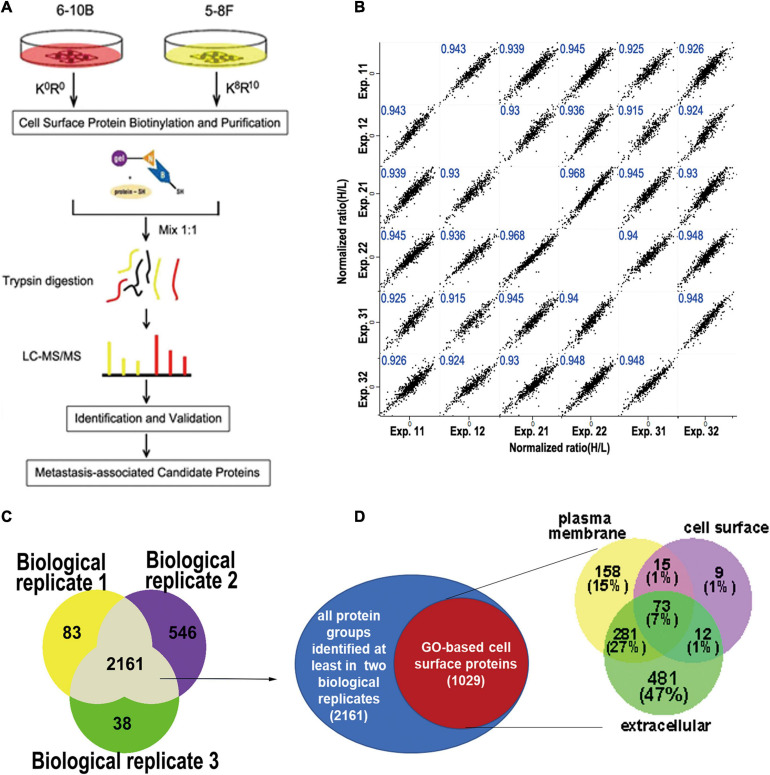
Experimental workflow for cell surface labeling and preparation for mass spectrometry. **(A)** Workflow of SILAC mass spectrometry-based analysis of the cell surface proteome, where 6-10b and 5-8F cells were labeled with “light” and “heavy” amino acids, respectively. **(B)** Scatter plot of protein ratio (5-8F vs. 6-10B) between the biological and technical replicates of the cell surface proteome. The Pearson correlation coefficient is presented in blue at the top of each plot. **(C)** Venn diagram showing the number of identified proteins from three biological replicate experiments. **(D)** Identified proteins grouped by their annotated subcellular localization (UniProtKB). Surface-exposed proteins represented by their annotated detailed categories are shown in the Venn diagram.

To minimize identification of non-surface-exposed proteins that had been labeled with Sulfo-NHS-SS-Biotin, we performed 3 biological replicate experiments for labeling and enrichment of cell surface proteins to examine the repeatedly enriched proteins which were more likely to be surface-exposed proteins (Figure 1A). In total, six experimental datasets were acquired. We calculated all pairwise Pearson correlations between these datasets. As shown in Figure 1B, the minimum Pearson correlation coefficient was 0.915, which demonstrates that the reproducibility of protein quantification was high at both the technical and biological level.

To increase the confidence of surface protein identification, we overlapped the three biological experimental datasets, and the proteins that were identified in at least two biological replicates (2161 proteins) were selected for subsequent analysis (Figure 1C). Of the 2161 proteins, 1029 proteins (47.6%) were annotated as plasma membrane, cell surface or extracellular region. As shown in Figure 1D, more than half (53%) of these proteins belonged in the PM or cell surface, which included transmembrane proteins such as integrins, cell adhesive molecules, receptors, and ion channels (Supplementary Table 1). The remaining (47%) proteins were membrane-associated proteins or interacted with the cell membrane according to GO annotation.

### Analysis and Validation of Differentially Expressed Proteins

**FIGURE 2:**
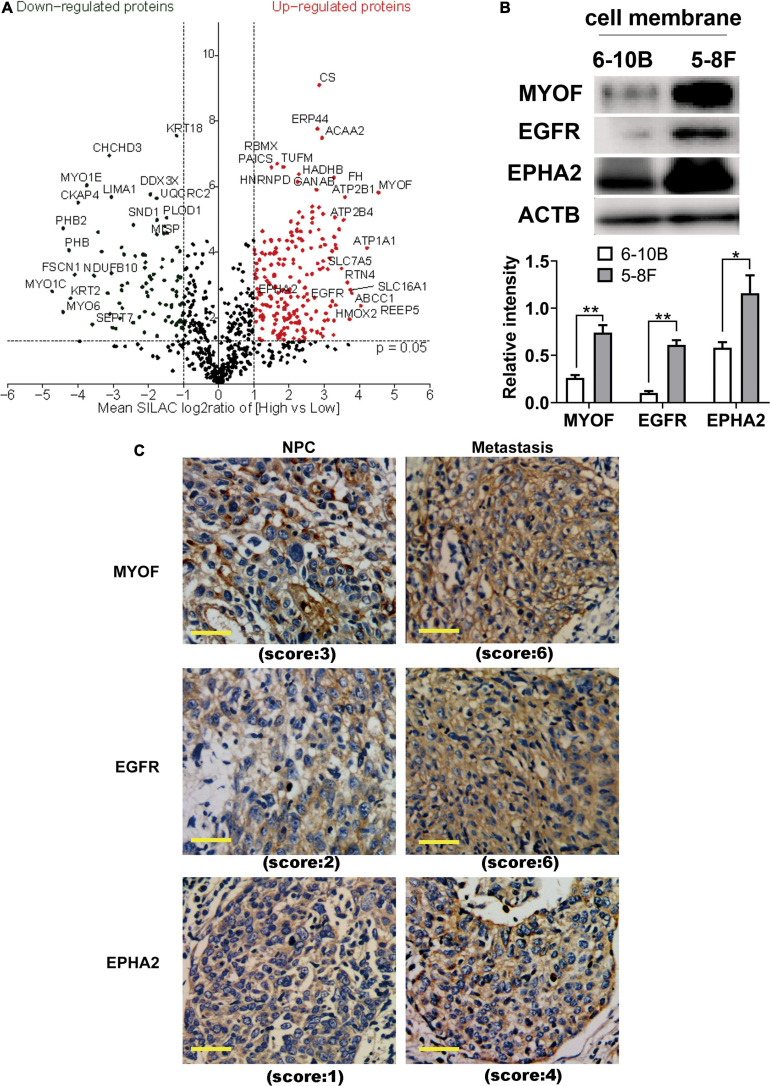
Identification and evaluation of significantly altered proteins between the 6-10B and 5-8F cell lines. **(A)** Volcano plot indicating significantly altered surface-exposed proteins identified in the datasets. Log-transformed *P*-values (*t*-test) against log-transformed fold change in abundance between 6-10B and 5-8F cells. **(B)** Western blot analysis evaluating the expression of proteins that reside in the plasma membrane. The density of the bands was analyzed by using NIH ImageJ software and normalized by the arbitrary units of b-actin. Data are the means ± SDs of 3 experiments. **p < 0.05 and **p < 0.01. **(C)** Representative immunohistochemistry (IHC) results showing increased detection of the indicated proteins in NPC with metastasis compared with NPC without metastasis.

To identify and quantify differentially expressed proteins between the two cell lines, we performed three biological replicate experiments. A one sample *t*-test was used to evaluate the significance of differences. Of the 1029 proteins, 294 proteins were significantly differentially expressed (fold change > 2, *p*-Value < 0.05, Figure 2A, Supplementary Table 2). Among these differentially expressed proteins, 206 proteins (93 plasma membrane proteins, 41 cell surface proteins and 72 extracellular proteins) were upregulated in the high metastatic NPC cell line (Table 1). The remaining 88 proteins (48 plasma membrane proteins, 5 cell surface proteins and 35 extracellular proteins) were downregulated in the high metastatic cell line. Table 2 shows the top 20 up- and downregulated proteins and their important information for identification, such as score, identified peptides, ratio, *p*-Value and subcellular location.

**TABLE 1:** The number of upregulated or downregulated proteins and their subcellular location.

	**Total**	**Subcellular location**		
		**Cell Surface**	**Plasma Membrane**	**Extracellular**
	294	46	141	107
Upregulation	206	41	93	72
Downregulation	88	5	48	35

**TABLE 2:** Top 20 list of up- and downregulated proteins in 5-8F vs. 6-10B cells.

**Symbol**	**Protein name**	**Score**	**Peptides**	**Log2(MR)**	***P*-value**	**PM**	**CS**	**SR**	**EC**
**Up-regulated proteins**									
MYOF	Myoferlin	323.31	146	4.54	1.50E–06	+			+
ATP1A1	Sodium/potassium-transporting ATPase subunit alpha-1	323.31	62	4.22	7.34E–05	+			+
ABCC1	Multidrug resistance-associated protein 1	266.82	37	3.78	1.74E–03	+			+
SLC16A1	Monocarboxylate transporter 1	206.15	4	3.74	1.39E–03	+			+
HMOX2	Heme oxygenase 2	323.31	20	3.72	1.09E–02	+			
RTN4	Reticulon-4	285.14	15	3.65	8.20E–04	+			+
ATP2B1	Plasma membrane calcium-transporting ATPase 1	323.31	53	3.59	2.08E–06	+			+
ATP2B4	Plasma membrane calcium-transporting ATPase 4	323.31	24	3.55	1.02E–05	+			
SLC7A5	Large neutral amino acids transporter small subunit 1	247.22	7	3.49	3.11E–04	+			+
SLC1A5	Neutral amino acid transporter B(0)	284.44	15	3.34	7.46E–05	+			+
ATP1B3	Sodium/potassium-transporting ATPase subunit beta-3	107.89	14	3.33	3.99E–03	+			+
PON2	Serum paraoxonase/arylesterase 2	86.501	3	3.29	3.36E–05	+			+
HTRA2	Serine protease HTRA2	26.469	10	3.28	1.18E–02	+			
GNA13	Guanine nucleotide-binding protein subunit alpha-13	26.947	11	3.26	3.27E–02	+			+
EGFR	Epidermal growth factor receptor	323.31	49	3.23	3.04E–03	+	+	+	+
PVR	Poliovirus receptor	165.18	8	3.17	4.38E–03	+	+		+
SLC2A1	Solute carrier family 2	57.723	6	3.15	1.50E–04	+			+
IGF1R	Tyrosine-protein kinase receptor	17.675	11	3.1	1.14E–02	+		+	
GOT2	Aspartate aminotransferase	300.24	20	3.05	7.17E–03	+			+
ICAM1	Intercellular adhesion molecule 1	226.69	19	3.02	5.84E–05	+	+		+
**Down-regulated proteins**									
MYO1C	Unconventional myosin-Ic	323.31	64	−4.73	1.55E–03	+			+
MYO6	Unconventional myosin-VI	323.31	61	−4.42	6.56E–03	+			+
PHB2	Prohibitin-2	323.31	29	−4.42	1.85E–05		+		+
PHB	Prohibitin	323.31	27	−4.25	8.51E–05	+	+		+
CKAP4	Cytoskeleton-associated protein 4	323.31	45	−3.99	3.06E–06	+			+
SEPT	Septin-7	203.21	17	−3.59	1.60E–02	+			+
SEPT2	Septin-2	323.31	19	−3.43	2.39E–05	+			+
MYO1B	Unconventional myosin-Ib	323.31	41	−3.15	1.73E–03	+			+
LIMA1	LIM domain and actin-binding protein 1	323.31	61	−3.05	2.04E–06	+			
ABCG2	ATP-binding cassette sub-family G member 2	41.72	9	−2.93	2.07E–02	+			
CORO1C	Coronin-1C	167.2	25	−2.76	7.03E–05	+			
IQGAP1	Ras GTPase-activating-like protein IQGAP1	323.31	102	−2.69	1.16E–04	+			+
KTN1	Kinectin	323.31	70	−2.59	8.49E–04	+			
MAP4	Microtubule-associated protein	179.13	18	−2.43	1.39E–02	+			+
ALPP	Alkaline phosphatase	323.31	23	−2.42	1.45E–05	+	+		
SLC25A11	Mitochondrial 2-oxoglutarate/malate carrier protein	111.51	12	−2.37	1.29E–04	+			
SERBP1	Plasminogen activator inhibitor 1 RNA-binding protein	202.11	9	−2.25	9.35E–03	+			+
LMO7	LIM domain only protein 7	323.31	44	−2.16	1.15E–04	+			
MAP7	Ensconsin	21.18	10	−2.11	2.70E–02	+			
CAPRIN1	Caprin-1	106.87	9	−2.1	6.40E–03	+			

*The listed proteins were analyzed with multiple biological repeats (heavy high metastatic NPC cell line: light low metastatic NPC cell line). For each protein, the following information is shown: adjusted *P*-values, logarithm of median ratio (high/low), number of quantified razor peptides plus unique peptides in a replicate. For identification, the peptide and protein FDRs were both set to 1% when using MAXQUANT. Peptides, Razor + unique peptides; MR, median ratio; PM, plasma membrane; CS, cell surface; SR, signaling receptor; EC, extracellular.*

In order to validate the proteomic results, three differentially expressed cell surface proteins, the most upregulated protein MYOF, and two other typical plasma proteins, EGFR and EPHA2, were selected for western blotting and immunohistochemistry analysis (Figures 2B,C). As shown in Figure 2B, immunoblots of MYOF, EGFR and EPHA2 revealed marked upregulation of these proteins in the membrane fraction, which were consistent with the quantitative proteomic measurements. Higher expression of these proteins was also detected in NPC tissues with metastasis than in those without metastasis.

### Bioinformatic Analysis of Differentially Expressed Proteins

**FIGURE 3:**
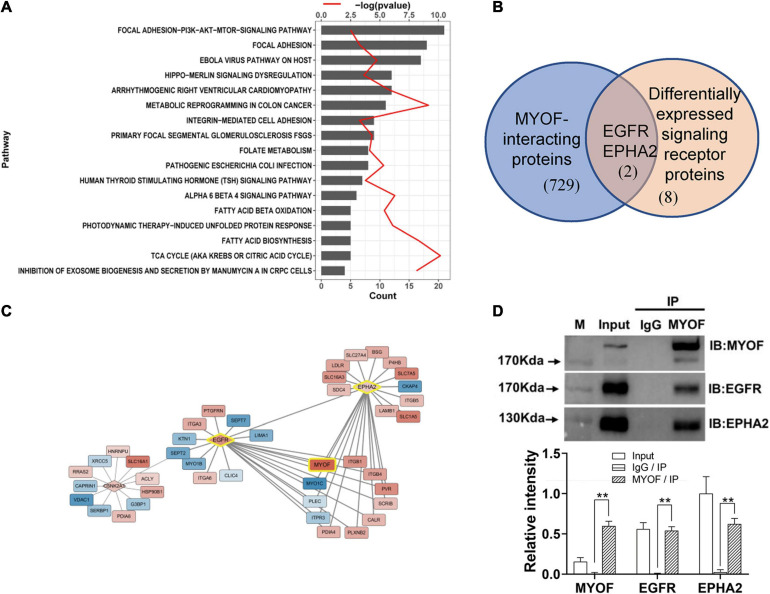
Signaling pathway enrichment analysis and interaction network analysis of DEPs **(A)** Enriched Wikipathway for differentially expressed surface-exposed proteins in 5-8F cell lines vs. 6-10B cell lines. **(B)** Overlapping of MYOF-interacting proteins and differentially expressed signaling receptors in 5-8F vs. 6-10B cells. MYOF-interacting proteins were retrieved using Harmonizome. **(C)** Partial interaction network of differentially expressed proteins focused on the interaction of MYOF, EGFR and EPHA2. **(D)** The interactions of MYOF with EGFR and EPHA2 were confirmed by Co-IP assay. Co-IP using anti-MYOF and negative control antibodies were performed in 5-8F cells. Western blots for all three proteins were performed. The density of the bands was analyzed by using NIH ImageJ software and normalized by the arbitrary units of the band of the input of EphA2. Data are the means ± SDs of 3 experiments. **p < 0.05 and **p < 0.01.

To investigate the biological function of the DEPs, the signaling pathways involved were investigated via GSEA analysis. Pathway enrichment analysis showed that the DEPs were primarily involved in biological processes, such as PI3K-AKT pathway, focal adhesions, virus-host interaction, integrin signaling, and metabolic reprogramming (Figure 3A), some of which are well known to be associated with metastasis. Of the 294 DEPs, 29 were involved in the Focal Adhesion-PI3K-mTOR (FAK-PI3K-mTOR) signaling pathway (Supplementary Figure 1). Intriguingly, all the plasma membrane signaling receptors among the DEPs were involved in this signaling pathway. These plasma membrane receptors, including 3 receptor tyrosine kinases and 7 integrin family members, are compiled in Table 3. These findings suggest that the FAK-PI3K-mTOR pathway might play a substantial role in NPC metastasis.

**TABLE 3:** Dysregulated signaling receptors in 5-8F vs. 6-10B cells.

**Symbol**	**Protein name**	**FAK-PI3K-mTOR**	**Score**	**Peptides**	**Log2(MR)**	**P-value**	**PM**	**CS**	**SR**
EGFR	Epidermal growth factor receptor	+	323.31	49	3.23	3.04E–03	+	+	+
IGF1R	Tyrosine-protein kinase receptor	+	17.675	11	3.1	1.14E–02	+		+
ITGB1	Integrin beta-1	+	310.22	28	2.95	3.91E–05	+	+	+
ITGB4	Integrin beta-4	+	323.31	78	2.6	2.33E–05	+	+	+
ITGA2	Integrin alpha-2	+	323.31	33	2.44	4.13E–02	+	+	+
ITGA3	Integrin alpha-3	+	219.91	19	2.29	1.27E–02	+	+	+
ITGA5	Integrin alpha-5	+	323.31	22	2.14	6.58E–03	+	+	+
ITGB5	Integrin beta-5	+	283.75	18	1.73	1.59E–04	+	+	+
ITGA6	Integrin alpha-6	+	323.31	37	1.61	1.10E–03	+	+	+
EPHA2	Ephrin type-A receptor 2	+	323.31	44	1.45	2.16E–03	+	+	+

Next, we constructed a protein interaction network using Harmonizome. In total, 731 proteins that interacted with MYOF were acquired from the Harmonizome datasets, among which 65 proteins were present in the DEP dataset (Supplementary Figure 2). Interestingly, two of the 10 differentially expressed plasma membrane receptors, EGFR and EPHA2, were among the 65 proteins (Figure 3B). An interaction network between the DEPs was assembled also from the Harmonizome datasets (Supplementary Figure 3). A partial interaction network focused on the interaction of MYOF, EGFR and EPHA2 is presented in Figure 3C. We can observe that only EGFR and EPHA2 have direct interaction with MYOF. We subsequently confirmed the interactions of MYOF with EGFR and EPHA2 using co-immunoprecipitation assay (Figure 3D). For further evaluating whether the interactions of MYOF with EGFR and EPHA2 are shared across tumor types, we analyzed MYOF co-expression with the members of common cell membrane receptor kinase (RTK) family from the proteomic data of 375 cancer cell lines from 22 lineages in CCLE. It is intriguing that EGFR and EPHA2 are the most related protein of MYOF and the correlation coefficients are 0.68 and 0.61, respectively (Supplementary Figure 5).

### MYOF Silencing Inhibits the Malignant Phenotype of NPC Cells

To characterize the function of MYOF in NPC, we depleted MYOF by stably transducing the high metastatic 5-8F cells with MYOF shRNA-expressing lentivirus.

**FIGURE 4:**
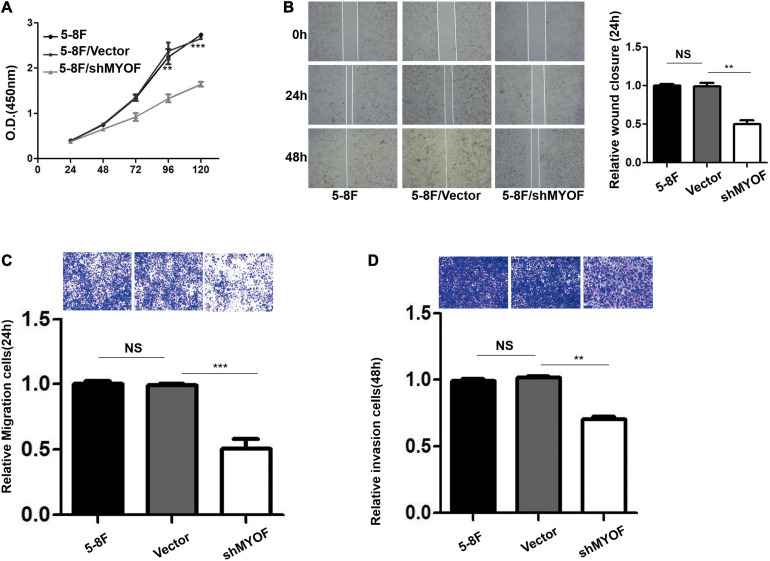
MYOF silencing reduces the malignant phenotype of 5-8F cells. **(A)** Cellular proliferation of control and MYOF knockdown 5-8F cells when passaged for the indicated days. Independent experiments performed in triplicate (**p < 0.01 and ***p < 0.001, respectively, Students *t*-test). **(B)** Wound-healing assay performed with Vector- or shMYOF-transfected 5-8F cells. Representative images acquired at the indicated time points are shown. The unhealed area was measured in three independent experiments (**p-value < 0.01). **(C)** Control or MYOF knockdown 5-8F cells were subjected to Transwell migration assays in three independent experiments. Migratory cells were counted under a microscope (**p < 0.01). **(D)** Control or MYOF knockdown 5-8F cells were subjected to Transwell invasion assays (with Matrigel). Invasive cells were counted under a microscope (**p < 0.01).

The shRNA vectors were constructed and yielded a >50% reduction in MYOF protein levels (Supplementary Figure 4). Compared with the control cells, MYOF depletion significantly reduced cellular proliferation rates by 50% (Figure 4A). We further investigated whether MYOF knockdown has an impact on migratory and invasive behavior. Our data indicated that the migration of 5-8F cells was severely inhibited in a wound-healing assay (Figure 4B). Likewise, the migration of 5-8F cells in a Transwell assay was significantly reduced (Figure 4C). In terms of invasion, a nearly 50% reduction in infiltration rate in Transwell invasion assays was observed in MYOF-depleted cells compared with control cells (Figure 4D). To extend our findings to tumor tissues, we investigated whether the protein level of MYOF was correlated with metastasis in surgically resected human NPC specimens. IHC was performed on samples from 50 cases of stage I–V NPC. The immunohistochemical staining scores indicated that the expression levels of MYOF were significantly higher in NPC with lymph node metastasis than without metastasis (*p* = 0.006, Table 4). Likewise, the expression of MYOF was also higher in NPC with distant metastasis than without metastasis (*p* = 0.003, Table 4). These results support the notion that the MYOF expression levels are associated with NPC metastasis potential. In summary, these results demonstrated that high expression of MYOF contributes to the malignant phenotype of metastatic NPC cells.

**TABLE 4:** Association between MYOF expression and clinicopathological characteristics in 50 nasopharyngeal carcinoma cases.

**Item**	**Number**	**MYOF**		**P-value**
		**Low (0-4)**	**High (5-8)**	
Gender				P = 0.374
Male	28	13	15	
Female	22	13	9	
Age(y)				P = 0.441
≥50	27	17	10	
<50	23	12	11	
Primary tumor(T) stages				P = 0.395
T1-2	23	10	13	
T3-4	27	15	12	
Lymph nodes metastasis				P = 0.006**
N0	22	14	8	
N1-N3	28	7	21	
Distant metastasis				P = 0.003**
M0	31	20	11	
M1	19	4	15	

**p < 0.01 determined by χ^2^-test, N0 vs. N1-3, and M0 vs.M1.

### MYOF Knockdown Interferes With Membrane Signaling Receptor Activity and EMT in NPC Cells

**FIGURE 5:**
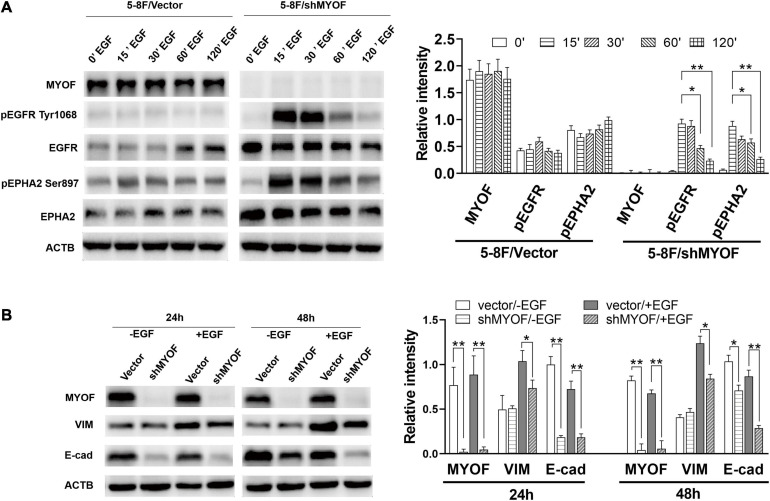
Myoferlin knockdown influences membrane receptor activity and EMT in NPC cells. **(A)** Western blot analysis of time-dependent expression and phosphorylation level of EGFR and EPHA2 following EGF stimulation and myoferlin knockdown. **(B)** MYOF knockdown inhibited EGF-induced VIM expression. The density of the bands was analyzed by using NIH ImageJ software and normalized by the arbitrary units of b-actin. Data are the means ± SDs of 3 experiments. *p < 0.05 and **p < 0.01.

In order to clarify the potential mechanism behind the knockdown of MYOF suppressing the malignant phenotype, we investigated whether MYOF knockdown can influence EGFR or EPHA2 activity in NPC cells. As shown in Figure 5A, upon EGF stimulation, prominent phosphorylation of the Y1068 residue was observed in MYOF-depleted NPC cells, which led to sustained pEGFR activation. Meanwhile, total EGFR expression levels were increased in comparison with those in vector-transfected cells. These findings demonstrate that MYOF influences EGFR and its phosphorylation-mediated activation status upon EGF stimulation. Previous studies have shown that EGF treatment promotes EMT-like morphological changes in all NPC cell lines (Yip and Seow, 2012). Increasing evidence indicates that EGF activate the EMT process, which promotes cell migration, invasion and metastasis (Wang and Zhou, 2013). Therefore, we speculated that MYOF depletion might influence EMT. To clarify this issue, we examined the expression alteration of EMT markers in MYOF knockdown 5-8F cells upon EGF stimulation. As shown in Figure 5B, in the presence of EGF stimulus, blank vector-transfected 5-8F cells exhibited strong induction of vimentin (VIM) expression in parallel with a weak downregulation of E-cadherin (E-cad) at both 24 h and 48 h, whereas the strong induction of VIM was observed at 24 h and 48 h in shMYOF-transfected cells. Moreover, the knockdown of MYOF led to a reduction of EGF-induced VIM, particularly at 48 h. MYOF knockdown also reduced E-cad at both 24 h and 48 h upon EGF stimulus. Likewise, we observed that the knockdown of MYOF led to the increasing of total EPHA2 expression levels and sustained EPHA2 ligand-independent activation, which was evidenced by prominent phosphorylation of the S897 residue. These data suggest that MYOF might interact with the two RTKs, EGFR and EPHA2, thereby regulating their expression and activation in the presence of specific stimulus.

## Discussion

Nasopharyngeal carcinoma arises from the nasopharynx epithelium and has high metastatic potential. It has been reported that approximately one third of cases relapse locoregionally or distantly despite intensive, definitive treatment (Cao et al., 2017). Novel treatment strategies beneficial against recurrence or for advanced patients are an important research direction. For example, recently, targeted therapy has demonstrated favorable responses and treatment outcomes (Zhang et al., 2013). Proteomic analysis of differentially expressed cell surface proteins during metastasis has been shown to be an efficient tool for discovering target candidates (Li et al., 2011; Xiao and Chen, 2019).

In present study, we performed the combination of cell surface biotinylation and SILAC-based quantitative proteomics to identify cell surface proteins associated with NPC metastasis. we found that 2161 proteins were present in at least in two biological replicates. Among them, 1029 proteins were identified as cell surface proteins according to GO or UniProt annotation. The number of identified proteins in previous studies aimed at profiling cell surface proteins ranged from less than 400 to approximately 1000 (Lund et al., 2009; Kuhlmann et al., 2018). Therefore, our results are comparable with those of previous studies (Karhemo et al., 2012; Li Y. et al., 2019). To the best of our knowledge, the present study presents the first and largest cell surface proteome of NPC published thus far.

Subsequently, through quantitative proteomic analysis, we identified 294 significantly differentially expressed proteins from the quantified cell surface proteins. Bioinformatic analysis showed that these proteins are involved in multiple pathways, some of which are related to metastasis. For example, in colon cancer, IL13 bind its receptor to activate the FAK-PI3K-mTOR pathway, resulting in cancer metastasis (Bartolomé et al., 2015). Increasing evidence has demonstrated that the mTOR complexes mTORC1 and mTORC2 participate in regulation of cell motility, invasion and cancer metastasis (Zhou and Huang, 2011). In our dataset, the largest number of differentially expressed cell surface proteins were aggregated in this pathway. Moreover, all the differentially expressed cell membrane receptors identified in present study were also involved in this pathway. Based on these observations, it was suggested that the FAK-PI3K-mTOR signaling pathway might be the most important pathway in regulation of NPC metastasis.

Adhesion-related proteins play an important role in the process of metastasis. Some of the differentially expressed proteins, such as Actin-1 (ACTN1), Caveolin-1 (CAV1), and Fibronectin-1 (FN1), were enriched in the Focal adhesion pathway. The roles of these adhesion-related proteins in cancer metastasis are not entirely clear, and some are even controversial. For example, Yu et al. found that CAV1 can promote hepatocellular carcinoma cell progression and metastasis through the Wnt/β-Catenin pathway (Yu et al., 2014), whereas Trimmer et al. demonstrated that CAV1 suppressed tumor growth and metastasis in a murine model of cutaneous squamous cell carcinoma through modulation of MAPK/AP-1 activation (Trimmer et al., 2013). Even in HCC, the function of CAV1 as a tumor suppressor or promoter is still under debate (Yang et al., 2010). These data indicated that malignant cells from different cancer types have different CAV1 expression profiles, which suggests that CAV1 biological functions might depend on the context. In the present study, CAV1 was significantly downregulated in the high metastatic cell line, but its biological role deserves further investigation.

In the list of differentially expressed cell surface proteins, the plasma membrane protein MYOF exhibited the largest fold change. MYOF is a member of the Ferlin family involved in membrane fusion, membrane repair, and membrane trafficking. More and more evidences indicate that MYOF has important significance in clinical diagnosis and targeted cancer therapy (Zhu et al., 2019). However, little is known about its involvement in NPC development and progression. To investigate the function of MYOF in NPC metastasis, we knocked down MYOF in high metastatic 5-8F cells. Consistent with a previous study in breast cancer cells (Turtoi et al., 2013) and Clear-Cell Renal-Cell Carcinoma (Cox et al., 2020), MYOF knockdown markedly *in vitro* inhibit cell proliferation, migration and invasion. Indeed, previous studies demonstrated that MYOF depletion reduced tumor development in a xenograft model of human breast cancer (Turtoi et al., 2013). Moreover, loss of MYOF or pharmacological inhibition of MYOF reduces breast cancer metastasis in an experimental mouse model, which demonstrated that targeting MYOF may be a promising therapeutic strategy in MYOF-driven breast cancer (Zhang et al., 2018). Immunohistochemical analysis showed that MYOF upregulation was related to NPC metastasis. It was in consistent with the finding that MYOF upregulation in many tumors was associated with metastasis (Wang et al., 2013; Blomme et al., 2017; Gu et al., 2020).

Bioinformatic analysis predicted that MYOF can interact with EGFR and EPHA2, both of which were also among the differentially expressed cell surface proteins. There are no experimental evidences supporting the existence of these interactions. Therefore, we performed Co-IP assay for the first time to verify the interactions of MYOF with EGFR and EPHA2. Based on these observations, we speculated whether the role of MYOF in regulating metastasis is related to its interaction with EGFR or EPHA2. Through shRNA inference assays, we found that MYOF knockdown significantly impaired the expression and phosphorylation level of EGFR, which is consistent with the study of Turtoi et al. in breast cancer cells (Turtoi et al., 2013). EGFR is a well investigated RTK whose activation and signaling contribute to tumor initiation and development, including proliferation, invasion, and metastasis. EGF induced EMT mediates the role of EGFR in promoting metastasis. Our data showed that MYOF knockdown in 5-8F cells significantly inhibited the expression of the EMT markers VIM and E-cad. Therefore, these observations indicated that MYOF knockdown can impair the downstream signaling of EGFR. EGFR is a therapeutic target for multiple types of cancer including NPC (Zhang et al., 2015). However, current EGFR targeted therapies remain somewhat unsatisfactory in the clinic, which might be due to limited knowledge of the mechanism (Peng et al., 2018). An in-depth understanding of MYOF as a regulator of EGFR might contribute to improvement of EGFR targeted therapy.

Ephrin type-A receptor 2 is a member of the Eph RTK family, which plays an essential role in both normal development and disease (Ieguchi and Maru, 2019). Unlike other family members, EPHA2 has a unique feature. It has two activation mechanisms, namely, ligand-dependent and ligand-independent activation, which have distinctive biological effects (Miao et al., 2009). EPHA2 ligand-independent activation, characterized by phosphorylation at S897, has been reported to contribute to metastasis in many tumors, such as colon cancer (Dunne et al., 2016), glioma (Miao et al., 2015), prostate cancer (Tawadros et al., 2012), and NPC (Li J. Y. et al., 2019). Indeed, EphA2 has been evaluated as a drug target using multiple approaches, such as agonist antibodies in leukemia (Charmsaz et al., 2015) and small molecule inhibitors in triple-negative breast cancer (Song et al., 2017). Our results showed that MYOF knockdown has an impact on EPHA2 ligand-independent activation and thus is a regulator of EPHA2 activity. It is the first time reported that MYOF is a regulator of EPHA2 activity. However, the link between MYOF function in metastasis and its regulation of EPHA2 activity deserves further study, which will contribute to the development of new therapy targeting MYOF or improvement of therapies targeting EPHA2. In fact, a previous study supposed that, in addition to EGFR, other RTKs might be regulated by MYOF in various cell types (Turtoi et al., 2013). For example, it was reported that MYOF regulates VEGFR2 stability and function in endothelial cells (Bernatchez et al., 2007). Furthermore, our analysis based on previous pan-cancer proteomic data also demonstrated that EGFR and EPHA2 are the most positive correlated with MYOF across different types. It suggested that MYOF interacts with EGFR and EPHA2 and regulates their activities are universal mechanism by which MYOF exerts its biological functions. In present study, we found that, in addition to EGFR, EPHA2 is a RTK regulated by MYOF in NPC. Our finding verified the supposition and further supported that MYOF might be a potential therapeutic target in NPC.

## Conclusion

In summary, our study provides a repertoire of cell surface proteins potentially useful for elucidating the mechanisms behind NPC metastasis. Moreover, we demonstrated that MYOF, a regulator of EGFR and EPHA2 activity, might be a potential target for development of new therapies for NPC.

## Data Availability Statement

The original contributions presented in the study are included in the article/Supplementary Material, further inquiries can be directed to the corresponding author/s.

## Author Contributions

ZC and YC designed the experiments and conducted the study supervision. ML, FP, and GW performed the experiments and analyzed the data. ML, YC, and GW wrote the manuscript. XL helped to analyze the data. MS helped to perform the experiments. All authors read and approved the final manuscript.

## Conflict of Interest

The authors declare that the research was conducted in the absence of any commercial or financial relationships that could be construed as a potential conflict of interest.

## Footnotes

1 http://download.baderlab.org

2 https://amp.pharm.mssm.edu/Harmonizome/

3 https://www.pathvisio.org/

4 https://gygi.hms.harvard.edu/publications/ccle.html
