# Supplementary material for: Coupling of Cell Surface Biotinylation and SILAC-Based Quantitative Proteomics Identified Myoferlin as a Potential Therapeutic Target for Nasopharyngeal Carcinoma Metastasis
Source: Front Cell Dev Biol. 2021 Jun 9;9:621810. doi: 10.3389/fcell.2021.621810 (PMC8219959; doi:10.3389/fcell.2021.621810)
Supplement: Supplementary file 4 [file Table_4.DOCX]

**Supporting Information**

**Coupling of cell surface biotinylation and SILAC-based quantitative proteomics identified myoferlin as a potential cell surface biomarker for nasopharyngeal carcinoma metastasis**

Maoyu Li^1,2,^**^†^**, Fang Peng^2,^**^†^**, Guoqiang Wang^2,^**^†^**, Xujun Liang^2^, Meiying Shao^2^, Zhuchu Chen^1,2*^, Yongheng Chen^1,2,3, *^

^1^Department of Gastroenterology, Xiangya Hospital, Central South University, Changsha, Hunan 410008, P. R. China.

^2^NHC Key Laboratory of Cancer Proteomics, Xiangya Hospital, Central South University, Changsha, Hunan 410008, China

^3^ State Key Laboratory of Medical Genetics and College of Life Science, Central South University, Changsha, Hunan 410008, China

† These authors contributed equally to the paper as first authors.

* Corresponding author: Prof. Yongheng Chen (yonghenc@163.com) and Prof. Zhuchu Chen (chenzhuchu@126.com), NHC Key Laboratory of Cancer Proteomics, Xiangya Hospital, Central South University, Hunan 410008, China

Figure S1. FAK-PI3K-mTOR Wikipathway was significantly enriched in differentially expressed cell surface proteins. Pathvisio was used to visualize the ratio values onto biological pathways obtained from Wikipathway. Up-regulated proteins are marked in red and down-regulated in blue.

Figure S2. Network analysis of differentially expressed proteins. A network of 294 differentially expressed proteins interactions were retrieved from various databases using Harmonizome and visualized by Cytoscape. Each node represents a protein, while Nodes were color-coded by negative fold-change (blue) to positive fold-change (red) in NPC cells.

Figure S3. Network analysis of MYOF. A network of MYOF and its direct interacted differentially expressed proteins was retrieved from network in Figure S2 and visualized by Cytoscape.

Figure S4. Western blot analysis was used to reveal the percentage of protein knockdown. β-actin was used as the internal loading control. MYOF protein expression in cells expressing a scrambled shRNA (vector) was used as the control. Quantification of the Western blot showed that the higher knockdown efficiency was observed in shRNA when compared to scramble control.

Figure S5. Pearson correlation between MYOF expression and denoted RTK genes across 375 different CCLE carcinoma cell lines among 22 lineages. A-D: MYOF correlation with EGFR family members, E-K: MYOF correlation with EPHA subfamily members; K-O: with EPHB subfamily members; P-S: MYOF correlation with FGFR family members. T: Summary graph of MYOF correlation with representative RTKs

Table S1. All the proteins identified in the present study. The peptides and proteins were identified both with an FDR of 1% using MaxQuant.

Table S2. List of differentially expressed cell surface proteins between high and low metastatic NPC cells. The subcellular location annotation of each protein was acquired from UniProt.

Table S3. Wikipathways enrichment analysis of differentially expressed cell surface proteins between high and low metastatic NPC cells. Enrichment analysis was performed using GSEA.
