# Supplementary material for: Coupling of Cell Surface Biotinylation and SILAC-Based Quantitative Proteomics Identified Myoferlin as a Potential Therapeutic Target for Nasopharyngeal Carcinoma Metastasis
Source: Front Cell Dev Biol. 2021 Jun 9;9:621810. doi: 10.3389/fcell.2021.621810 (PMC8219959; doi:10.3389/fcell.2021.621810)
Supplement: Supplementary file 5 [file Data_Sheet_1.PDF]

Figure S1

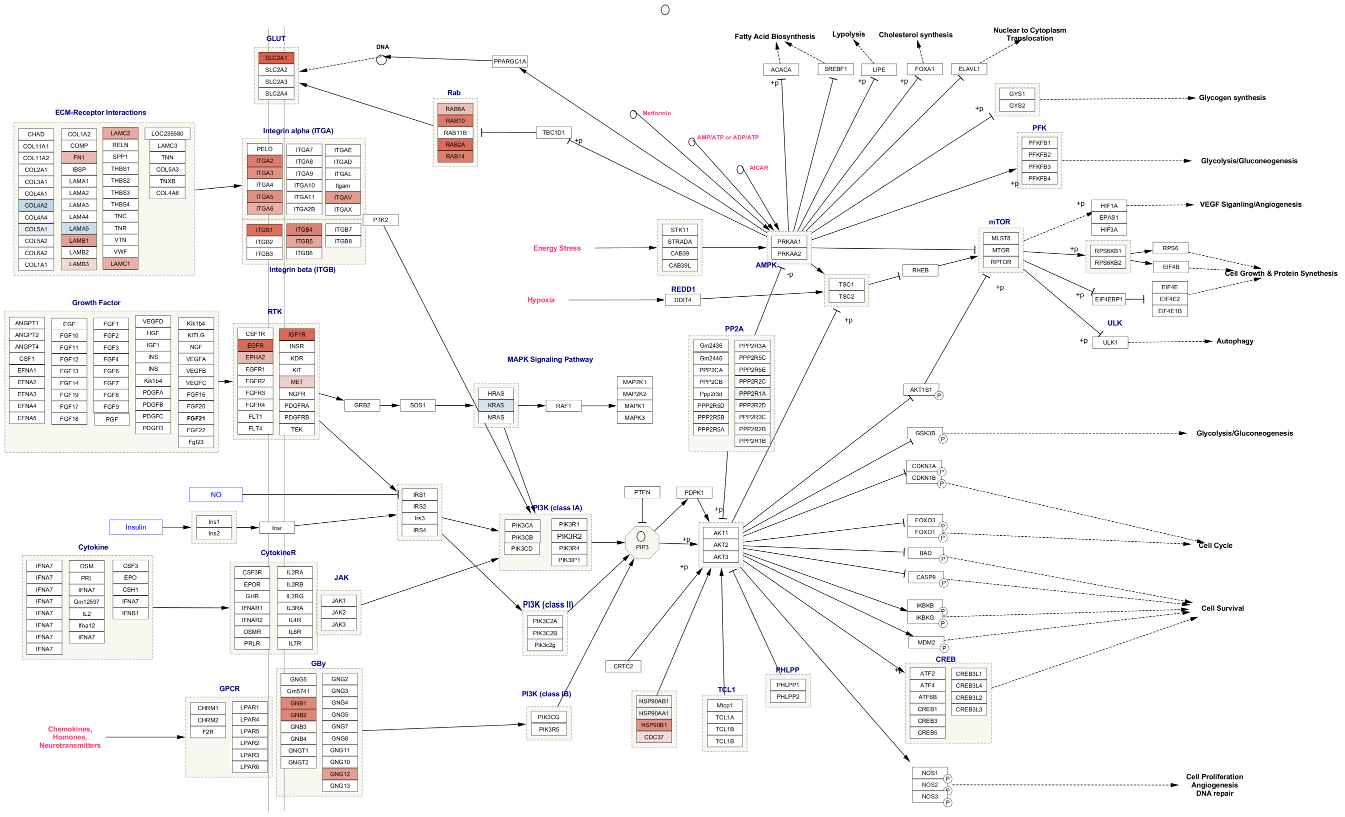

Figure S1. FAK-PI3K-mTOR Wikipathway was significantly enriched in differentially expressed cell surface proteins. Pathvisio was used to visualize the ratio values onto biological pathways obtained from Wikipathway. Up-regulated proteins are marked in red and down-regulated in blue.
