# Supplementary material for: Coupling of Cell Surface Biotinylation and SILAC-Based Quantitative Proteomics Identified Myoferlin as a Potential Therapeutic Target for Nasopharyngeal Carcinoma Metastasis
Source: Front Cell Dev Biol. 2021 Jun 9;9:621810. doi: 10.3389/fcell.2021.621810 (PMC8219959; doi:10.3389/fcell.2021.621810)
Supplement: Supplementary file 6 [file Data_Sheet_2.PDF]

# Figure S2

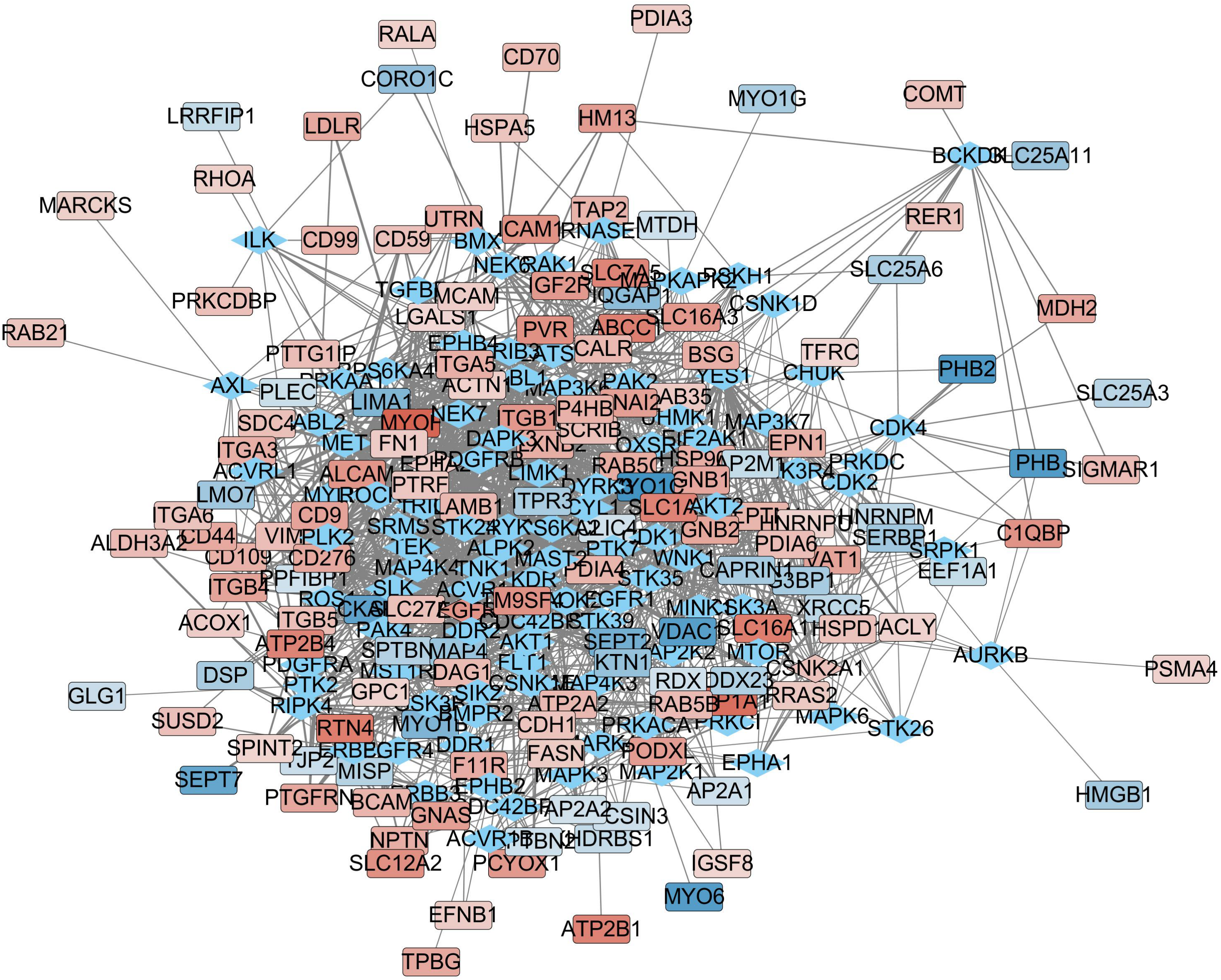


Figure S2. Network analysis of differentially expressed proteins. A network of 294 differentially expressed proteins interactions were retrieved from various databases using Harmonizome and visualized by Cytoscape. Each node represents a protein, while Nodes were color-coded by negative fold-change (blue) to positive fold-change (red) in NPC cells.
