# Supplementary material for: Coupling of Cell Surface Biotinylation and SILAC-Based Quantitative Proteomics Identified Myoferlin as a Potential Therapeutic Target for Nasopharyngeal Carcinoma Metastasis
Source: Front Cell Dev Biol. 2021 Jun 9;9:621810. doi: 10.3389/fcell.2021.621810 (PMC8219959; doi:10.3389/fcell.2021.621810)
Supplement: Supplementary file 7 [file Data_Sheet_3.PDF]

Figure S3

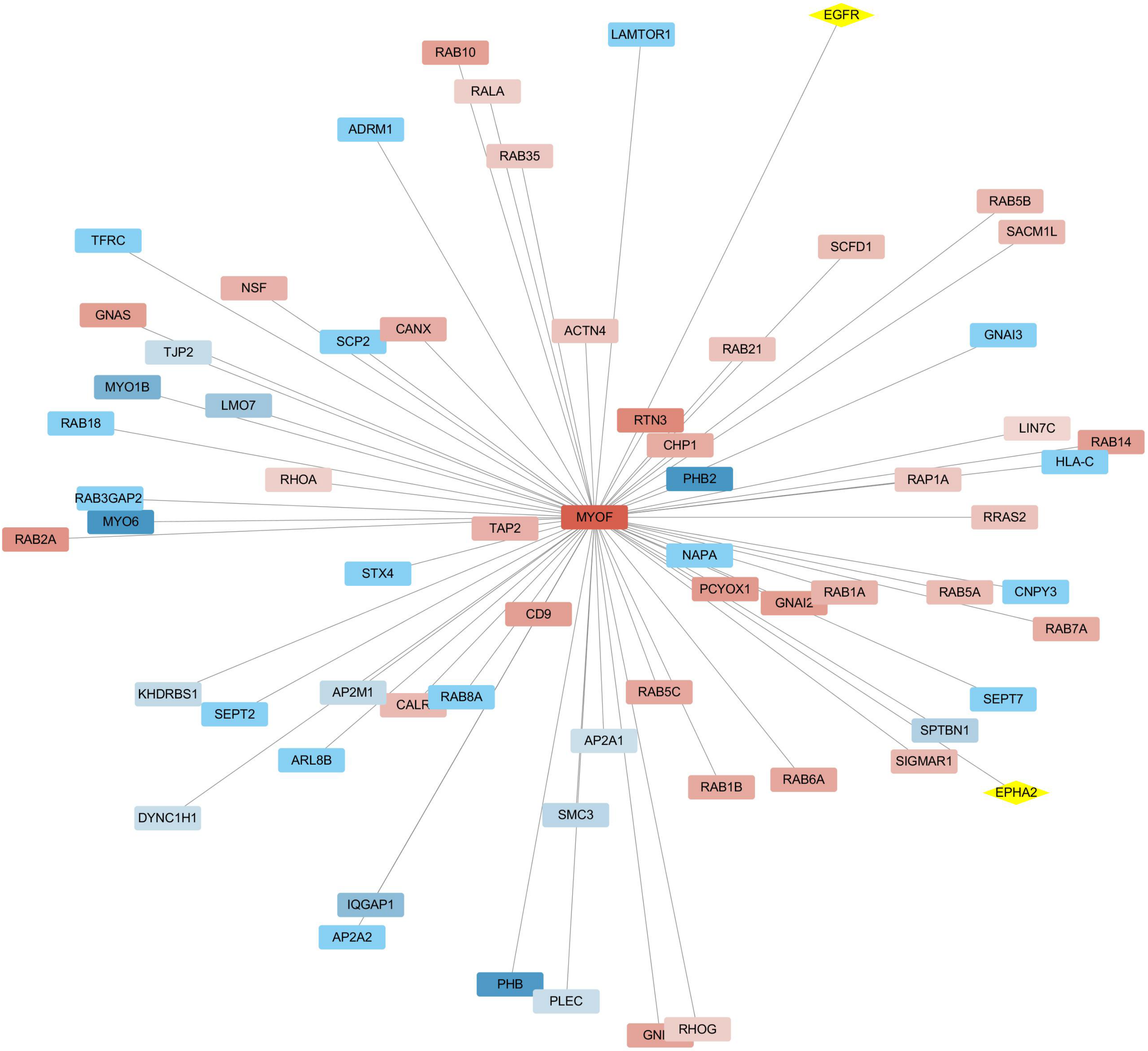

Figure S3. Network analysis of MYOF. A network of MYOF and its direct interacted differentially expressed proteins was retrieved from network in Figure S2 and visualized by Cytoscape.
