# Supplementary material for: Coupling of Cell Surface Biotinylation and SILAC-Based Quantitative Proteomics Identified Myoferlin as a Potential Therapeutic Target for Nasopharyngeal Carcinoma Metastasis
Source: Front Cell Dev Biol. 2021 Jun 9;9:621810. doi: 10.3389/fcell.2021.621810 (PMC8219959; doi:10.3389/fcell.2021.621810)
Supplement: Supplementary file 8 [file Data_Sheet_4.PDF]

**Figure S4**

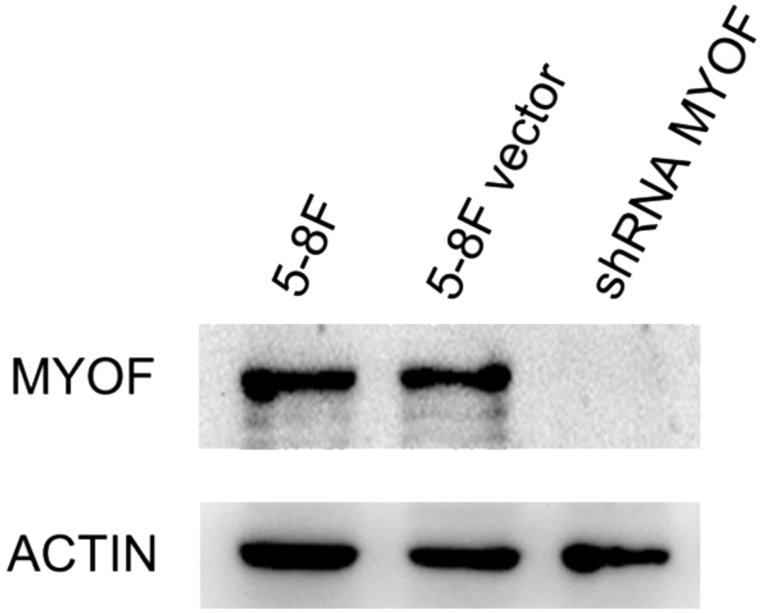


Figure S4. Western blot analysis was used to reveal the percentage of protein knockdown. β-actin was used as the internal loading control. MYOF protein expression in cells expressing a scrambled shRNA (vector) was used as the control. Quantification of the Western blot showed that the higher knockdown efficiency was observed in shRNA when compared to scramble control.
