# Supplementary material for: Coupling of Cell Surface Biotinylation and SILAC-Based Quantitative Proteomics Identified Myoferlin as a Potential Therapeutic Target for Nasopharyngeal Carcinoma Metastasis
Source: Front Cell Dev Biol. 2021 Jun 9;9:621810. doi: 10.3389/fcell.2021.621810 (PMC8219959; doi:10.3389/fcell.2021.621810)
Supplement: Supplementary file 9 [file Data_Sheet_5.PDF]

Figure S5

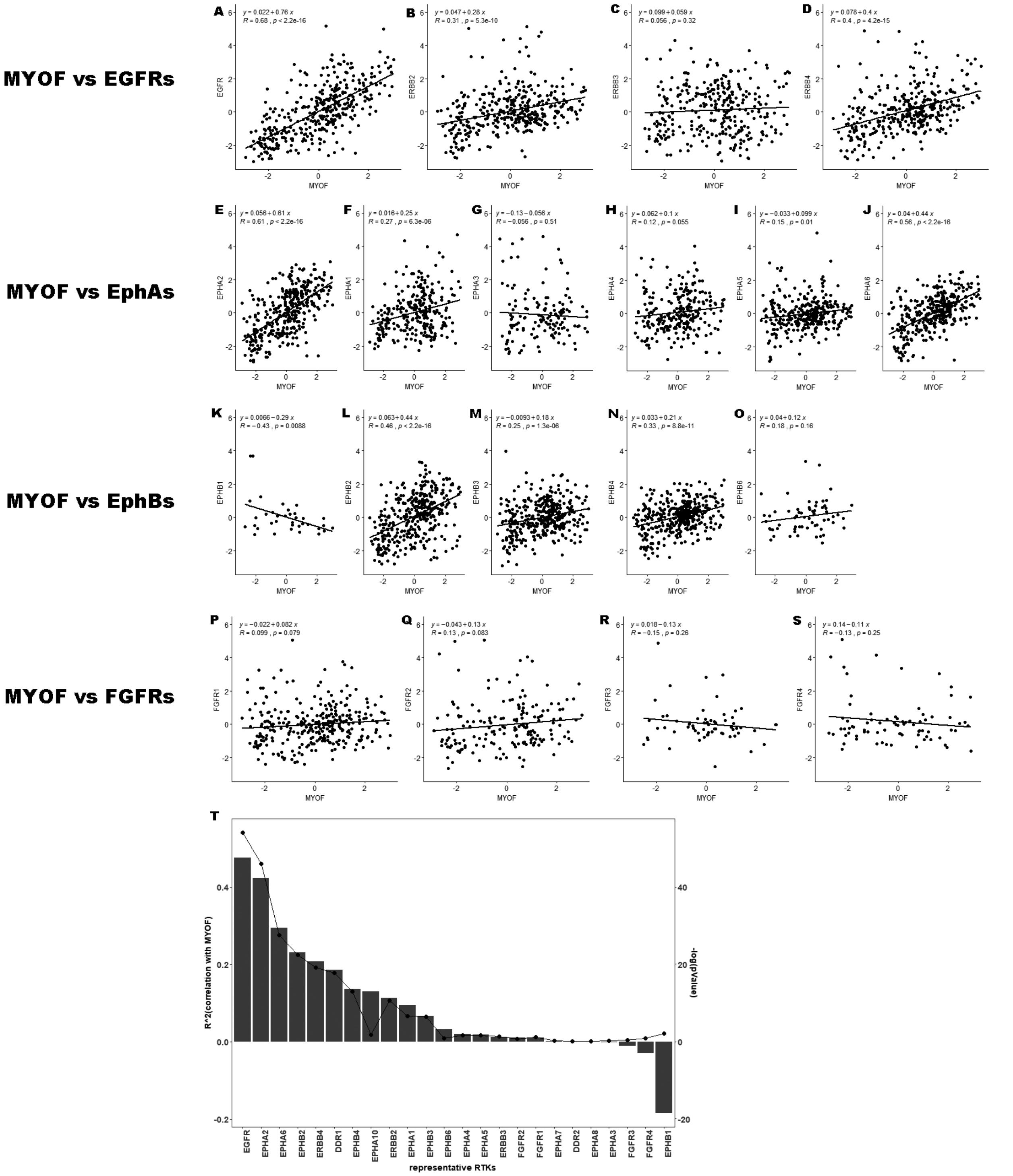

Figure S5. Pearson correlation between MYOF expression and denoted RTK genes across 375 different CCLE carcinoma cell lines among 22 lineages. A-D: MYOF correlation with EGFR family members, E-K: correation with EPHA subfamily members; K-O: with EPHB subfamily members; P-S: MYOF correlation with FGFR family members. T: Summary graph of MYOF correlation with representative RTKs
